# Impact of Renal Failure on F18-FDG PET/CT Scans

**DOI:** 10.3389/fonc.2017.00155

**Published:** 2017-07-21

**Authors:** Vishwajit Kode, Holly Karsch, Medhat M. Osman, Razi Muzaffar

**Affiliations:** ^1^Saint Louis University School of Medicine, Saint Louis, MO, United States; ^2^Division of Nuclear Medicine Technology, Saint Louis University Hospital, Saint Louis, MO, United States; ^3^Division of Nuclear Medicine, Department of Radiology, Saint Louis University, Saint Louis, MO, United States

**Keywords:** PET/CT, renal failure, ESRD, FDG, PET

## Abstract

**Objective:**

The current guidelines for 2-deoxy-2-[18F]fluoro-d-glucose PET/CT scanning do not address potential inaccuracies that may arise due to patients with renal failure. We report a retrospective analysis of standard uptake values (SUVs) in patients with and without renal failure in order to warrant a protocol adjustment.

**Methods:**

Patients were matched based on age, gender, and BMI all of which are potential effectors on observed SUV. Thirty patients were selected with clinically diagnosed renal failure, of which 12 were on dialysis. All 30 patients had age, gender, and BMI control matches. Blood urea nitrogen and creatinine levels were measured within 1 month of the scan to assess renal failure. PET/CT scans for both the renal failure patients and controls were performed 60 min after FDG injection. SUVs were measured by placing circular regions of interest in the right hepatic lobe (LSUV) and left psoas muscle (PSUV).

**Results:**

For the 30 renal failure patients, the mean LSUV was 2.77 (SD = 0.57) and PSUV was 1.43 (SD = 0.30) while the controls had mean LSUV 2.74 (SD = 0.50) and PSUV 1.42 (SD = 0.37). The SUVs from both the liver and psoas muscle were not significantly different between the renal failure patients and the normal controls with *p* values >0.05. In addition, dialysis and gender also had no effect on SUVs.

**Conclusion:**

Our data suggest that renal failure patients do not require an adjustment in protocol and the standard protocol times should remain.

## Introduction

According to the Center for Disease Control, the ninth leading cause of death in the United States is kidney disease with more than 47,000 deaths each year (1). Kidney disease is a prevalent and growing problem in the United States with over 26 million American adults afflicted (2). The two leading causes of kidney disease are high blood pressure and diabetes, both of which are rising (3). In addition to kidney disease, many of these patients have various other co-morbidities such as cancer. Consequently, there is a large overlap in these patient populations. In 2015, an estimated 1.7 million FDG PET/CT scans were performed while over 1.6 million cancer cases were newly diagnosed (4, 5). Therefore, it is important to evaluate the effects of renal failure on the biodistribution of FDG in FDG PET/CT scans.

Renal failure is defined as an 85–90% loss of kidney function with a glomerular filtration rate less than 15 ml/min/1.73m^2^. Treatments for renal failure include kidney transplants, hemodialysis, and peritoneal dialysis (6). It is hypothesized that patients with renal failure may require a greater uptake time during an FDG PET/CT exam than patients with normal kidney function due to the impaired distribution and clearance of FDG. Other nuclear medicine studies such as bone scanning have addressed the altered biodistribution caused by renal failure and recommend additional delayed imaging to allow for an improved target to background ratio (7). However, neither the US nor the European guidelines addresses the impact of kidney disease on PET/CT scanning and whether there should be an adjustment to the protocol (8, 9). The standard uptake time for most malignancies is 60 min after the injection of FDG (10). While this is standard for a patient with normal kidney function, it is believed that a patient with renal failure may need a longer uptake time to improve the target to background ratio as in bone scanning.

Currently, information regarding FDG clearance time in patients with renal failure is limited. However, it has been hypothesized that a patient with renal failure might need a greater uptake time to improve diagnostic accuracy (11). Currently, the only exceptions to the 60-min uptake time are breast, hepatocellular, prostate, and pancreatic cancers, which require a 90 min uptake. However, a study has been performed on patients with high creatinine and found that the FDG accumulated in the blood of these patients (12).

In this study, we hypothesized higher standard uptake values (SUVs) in the internal reference points in patients with renal failure compared to the age and gender matched controls which could imply a need for a change in protocol.

## Materials and Methods

### Patient Selection

Our Institutional Review Board approved this single institution, retrospective study, and the requirement to obtain informed consent was waived. We retrospectively reviewed 1,095 [18F]fluoro-d-glucose PET/CT scans of known cancer patients. The majority of patients were scanned from the vertex of the skull to the toes, as it is the standard of care in our institution. A log was kept for patients with clinically diagnosed renal failure. Exclusion criteria included patients with renal transplant, patients with primary liver or metastatic cancer in the liver, patients who did not have blood urea nitrogen or creatinine levels within 1 month of the scan and patients who were given a 90 min uptake time instead of the standard 60 min uptake. Thirty patients were selected with 12 of these patients on dialysis. However, this was not considered as a parameter for exclusion.

In order to minimize variance between a control group and the renal failure patients, we selected 30 controls with normal kidney function that matched each renal failure patient in BMI, age, and gender. In addition, BUN and creatinine levels were measured within 1 month of the scan. The BUN levels for the control group fell within the acceptable 7–26 mL/dL limit and the creatinine levels fell within the acceptable 0.6–1.2 mL/dL limit.

### PET/CT Scan

FDG PET/CT scans were acquired using PET/CT scanner (Gemini TF; Philips Medical Systems) with an axial co-scan range of 193 cm. Per institutional protocol, all patients were instructed to fast at least 4 h prior to receiving the radiopharmaceutical injection. Blood glucose level was <200 mg/dL in all patients. On the day of the exam, intravenous injection of 5.18 MBq/kg (0.14 mCi/kg) of FDG was administrated. Patients sat in a quiet room without talking for 60 min during the uptake phase prior to imaging.

### CT Scanning

The CT component of the PET/CT scanner has 64 multidetector helical CT with a gantry port of 70 cm. The parameters of CT detectors were set as follow for 20–21 bed acquisitions: 120–140 kV and 33–100 mAs (based on body mass index), 0.5 s per CT rotation, pitch of 0.9 and 512 × 512 matrix data were used for image fusion and the generation of the CT transmission map. The CT images were obtained without oral or IV contrast administration according to the standard PET/CT protocol at our institution.

### PET Scanning and Image Processing

The PET component of the PET/CT scanner is composed of lutetium-yttrium oxyorthosilicate-based crystal. Emission scans were acquired at 1–2 min per bed position. The FOV was from the top-of-head to the bottom of feet in the vast majority of patients. The three-dimensional (3D) whole-body (WB) acquisition parameters were 128 × 128 matrix and 18 cm FOV with a 50% overlap. Processing used the 3D Row Action Maximum Likelihood Algorithm method. Total scan time per patient was approximately 20–45 min.

### Data Analysis

PET/CT images were retrospectively evaluated on the Gemini TF extended brilliance workstation by board certified nuclear medicine physicians. Quantitative analysis of the data was done using SUV, standardized maximum uptake values. A 30 mm circular region of interest was used to record the liver SUV (LSUV) from the right hepatic lobe and psoas SUV (pSUV) from the left psoas muscle.

The median SUVs for both the control group and the renal failure group were compared using a Mann–Whitney *U* test. In addition, Mann–Whitney *U* test comparison was also done for the dialysis and non-dialysis patients using the same methodology. Statistics were completed using IBM SPSS Statistics, version 23. Statistical significance was set at *p* < 0.05.

## Results

**Figure 1:**
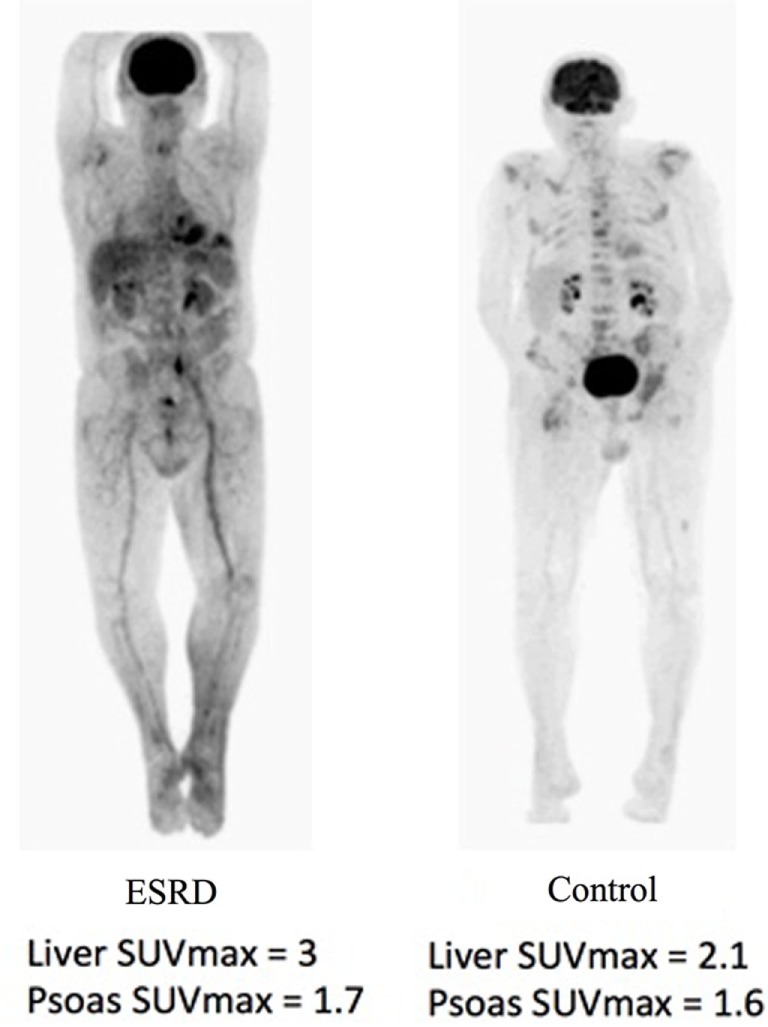
In a typical patient, the PET/CT will show an accumulation of the F18-FDG marker in areas that consume the most glucose, as well as the renal system in the body. As a result, areas of accumulation appear in the brain, kidneys, and bladder. The brain consumes much of the glucose and the kidneys filter the marker to the bladder. In the patient with renal failure, due to the slower filtration rate of blood, the areas of uptake seem to be increased within the soft tissue.

**Figure 2:**
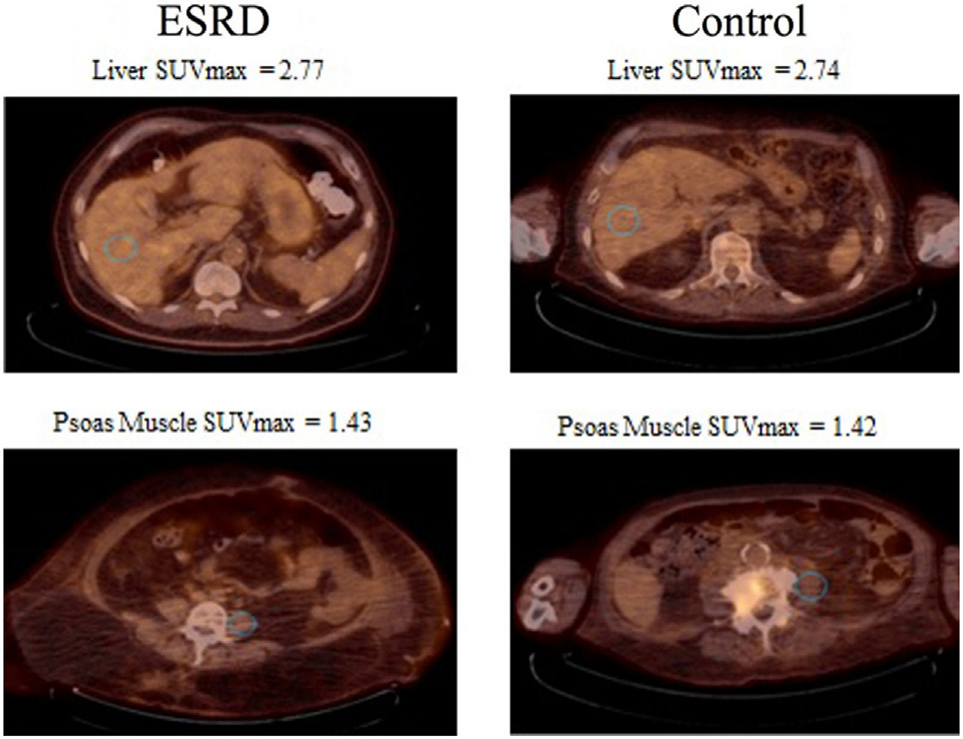
30 mm circular regions of interest (blue) were placed. The first in the right hepatic lobe and second in the left psoas muscle. In addition, the figure shows the mean standard uptake values (SUVs) between the controls and the renal failure patients.

The participant characteristics are presented in Table 1. For the 30 renal failure patients, the median LSUV 2.90 (min–max = 1.60–3.90) and PSUV 1.30 (min–max = 1.10–2.50) while the controls had median LSUV was 2.60 (min–max = 1.80–3.90) and PSUV was 1.35 (min–max = 0.90–2.80). The median SUVs from both the liver (*p* = 0.62) and psoas muscle (*p* = 0.57) were not significantly different between the renal failure patients and the normal controls (Table 2). In addition, the dialysis patients demonstrated median LSUV was 2.95 (min–max = 2.00–3.80) and mean PSUV was 1.30 (min–max = 1.01–1.90) and the non-dialysis patients had LSUV 2.75 (min–max = 1.60–3.90) and PSUV 1.30 (min–max = 1.10–2.50), differences between them were not significant (Table 3). Figure 1 illustrates the FDG distribution in a typical renal failure patient and control. Figure 2 demonstrates the circular region of interest on the liver and left psoas muscle.

**Table 1:** Patient characteristics.

	Overall (*n* = 60)	Renal failure (*n* = 30)	Matched controls (*n* = 30)	*p*-Value
Age, mean (SD)	68.93 (12.63)	68.17 (12.66)	69.14 (12.81)	0.90
Gender, *n* (%)				1.00
Male	42 (70.0)	21 (70.0)	21 (70.0)	
Female	18 (30.0)	9 (30.0)	9 (30.0)	
BMI, mean (SD)	26.66 (5.86)	26.82 (5.55)	26.50 (6.24)	0.84

**Table 2:** Median (min–max) liver and psoas muscle standard uptake values (SUVs) for renal failure and control patients.

	Renal failure (*n* = 30)	Matched controls (*n* = 30)	*p*-Value
Liver SUVmax	2.90 (1.60–3.90)	2.60 (1.80–3.90)	0.62
Psoas muscle SUVmax	1.30 (1.10–2.50)	1.35 (0.90–2.80)	0.57

**Table 3:** Median (min–max) liver and psoas muscle standard uptake values (SUVs) for renal failure patients on dialysis and control patients.

	Dialysis (*n* = 12)	Non-dialysis (*n* = 18)	*p*-Value
Liver SUVmax	2.95 (2.00–3.80)	2.75 (1.60–3.90)	0.20
Psoas muscle SUVmax	1.30 (1.10–3.50)	1.30 (1.10–2.50)	0.54

## Discussion

The basics of pharmacokinetics state that drugs which are cleared primarily by the kidneys will have altered biodistribution and will require dose adjustment with compromised renal function (13). It has been established that renal clearance is integral for FDG metabolism and with the increased rates of renal failure in America, it is essential the effects of renal failure on FDG uptake are studied. However, there is limited information in the US and European guidelines as well as the literature to assess impaired renal function on FDG PET/CT scans. We hypothesized that patients with renal failure would have slower clearance of FDG and would require a longer time for the tracer to be metabolized. Consequently, patients with renal failure and in need of an FDG PET/CT scan could require an altered uptake time that may be different from the uptake times of patients without renal failure.

In this study, we compared the SUVs of internal reference points of patients with renal failure to age, gender, and BMI-matched controls. We found the SUVs for these patients are not statistically different from the non-renal failure patients. The data to support this come from the *p*-value for renal failure patients and the control group for both liver and psoas muscle were >0.05. In addition, we also compared renal failure patients on dialysis to normal controls who were not on dialysis and they too had no significant difference in their SUVs. This was also indicated by a *p*-value >0.05. Therefore, we can conclude that the uptake time in patients with renal failure does not need to change to compensate for any altered biodistribution.

Our study is not without limitations. The retrospective nature of the study is a potential limitation. Also, imaging at different time intervals could give further verification if an increased uptake time would be statistically significant. A study of this nature was performed by Akers et al. (14) who investigated the effects of various degrees of compromised renal function on FDG uptake and clearance at multiple time points in normal tissues. They too concluded that compromised renal function did not affect clearance of background activity. However, some limitations of their study were their lack of controls, which were present in our study. Nevertheless, they also concluded that there is no need to alter standard uptake times for patients with renal failure.

The sample size of our study was relatively small with only 30 renal failure patients. Some models have shown an association between the severity of the renal failure and the inaccuracy of the SUVs (15). A larger sample size would allow for various stages and severities of renal failure. The severity could be an important factor due to the urine excretion rate. Since urine production has such a drastic impact on the amount of dosage excreted, it can be associated with the rates of delayed urine production between severe and mild renal failure. Lastly, there was no standardization for hydration status in our study before the PET/CT scanning.

With the limited information on the topic of renal failure affecting the uptake time for FDG, it is important that further studies are done to fully comprehend any affects that it may have on PET/CT scans. As the rates of renal failure increase and the increased utility of PET/CT, it is imperative that any factor affecting image quality be addressed to avoid inaccurate interpretations.

## Conclusion

Renal disease has not been found to have a significant impact on the FDG biodistribution in FDG PET/CT studies. Therefore, patients with renal failure do not require an adjustment to the protocol.

## Author Contributions

VK is the first author and wrote the major portion of the manuscript. HK performed the data collection and helped writing the manuscript as well as capturing figures. MO assisted in editing and writing the manuscript. RM assisted in writing, editing, and optimizing figures for the manuscript.

## Conflict of Interest Statement

The authors declare that the research was conducted in the absence of any commercial or financial relationships that could be construed as a potential conflict of interest.
